# Invasive lionfish reduce native fish abundance on a regional scale

**DOI:** 10.1038/srep32169

**Published:** 2016-08-31

**Authors:** Nicholas G. Ballew, Nathan M. Bacheler, G. Todd Kellison, Amy M. Schueller

**Affiliations:** 1 National Marine Fisheries Service, Southeast Fisheries Science Center, Beaufort Laboratory, 101 Pivers Island Road, Beaufort, NC 28516 USA

## Abstract

Invasive lionfish pose an unprecedented threat to biodiversity and fisheries throughout Atlantic waters off of the southeastern United States, the Caribbean, and the Gulf of Mexico. Here, we employ a spatially replicated Before-After-Control-Impact analysis with temporal pairing to quantify for the first time the impact of the lionfish invasion on native fish abundance across a broad regional scale and over the entire duration of the lionfish invasion (1990–2014). Our results suggest that 1) lionfish-impacted areas off of the southeastern United States are most prevalent off-shore near the continental shelf-break but are also common near-shore and 2) in impacted areas, lionfish have reduced tomtate (a native forage fish) abundance by 45% since the invasion began. Tomtate served as a model native fish species in our analysis, and as such, it is likely that the lionfish invasion has had similar impacts on other species, some of which may be of economic importance. Barring the development of a control strategy that reverses the lionfish invasion, the abundance of lionfish in the Atlantic, Caribbean, and Gulf of Mexico will likely remain at or above current levels. Consequently, the effect of lionfish on native fish abundance will likely continue for the foreseeable future.

The devil firefish (*Pterois miles*) and the red lionfish (*P. volitans*), both collectively referred to as lionfish in this study, are generalist piscivores that have invaded Atlantic waters off of the southeastern United States (hereafter abbreviated as SEUS), the Gulf of Mexico, and the Caribbean^1^,^2^. The lionfish invasion is thought to be the result of multiple lionfish release events that occurred via the aquarium trade during the late 1980s and early 1990s^3^,^4^. Once introduced, their growth rate, high fecundity, generalist foraging habits, and lack of predators led to their rapid spread^5^,^6^,^7^. The first assessment of lionfish densities in their non-native range, which was conducted in 2004, reported an average of 21 lionfish ha^−1^ across 17 locations off North Carolina^8^. By 2008, the maximum lionfish densities observed off North Carolina were approximately 450 lionfish ha^−1^, with mean densities of about 150 lionfish per hectare^9^. Today, lionfish densities exceed those of all but the most common predatory fish species on many reefs in the Caribbean and off of the SEUS^10^,^11^.

Experimental manipulations on patch reefs have demonstrated that lionfish can negatively affect native forage fish populations^12^,^13^. The introduction of a single lionfish into 4 m^2^ experimental patch reefs reduced recruitment of native prey fish by an average of 79% over a 5-week period^12^. When lionfish densities were controlled on larger experimental patch reefs (0.16–0.40 hectares) and over a longer time period (14 months), the abundance of prey fish at sites with 300 lionfish ha^−1^ was roughly 40% lower than the abundance of prey fish at sites with 30 lionfish ha^−1^ 10. Similar negative effects have also been documented at lionfish densities of 250 lionfish per hectare^14^. Further, modeling efforts have demonstrated that lionfish densities of roughly 250 ha^−1^ are sufficient to deplete prey fish populations faster than they can replenish themselves^14^.

The combination of results from lionfish density monitoring studies^8^,^9^ and experimental manipulations of lionfish densities^10^,^12^,^14^ suggest that lionfish have been negatively affecting the abundance of native fish in Atlantic waters off of the SEUS since the early- to mid-2000s. However, experimental manipulations of lionfish densities have focused on the effect of invasive lionfish near the upper bound of their natural densities (in their invaded range), have been short in duration (weeks to months), and have been conducted on relatively small spatial scales (5–30 km^2^) in localized areas off of the Bahamas^10^,^12^,^14^. Consequently, the impact of the ongoing lionfish invasion on native fish remains poorly understood. While one other study did investigate abundance changes in native reef fish in relation to lionfish, the study contained only 2 time points (2008 and 2010) and was also conducted in a localized area off of the Bahamas^15^. Additionally, the study lacked a non-invaded control group. Thus, studies that quantify the regional-scale impact of the lionfish invasion over multiple years are sorely needed.

The current study presents results on the abundance of tomtate (*Haemulon aurolineatum*) from North Carolina to Florida over the entire course of the lionfish invasion (1990–2014). Tomtate is a small grunt species that schools on reefs in Atlantic waters from Cape Cod, Massachusetts, to Brazil^16^,^17^. Within its range, tomtate is abundant on most reefs south of Chesapeake Bay and serves as prey to many economically important species such as snappers and groupers^16^. Tomtate is also a main dietary item of lionfish in waters off of the SEUS^18^. Thus, tomtate is a model species to use for an investigation into the impact of the lionfish invasion on native fish abundance. To conduct our investigation, we employed a Before-After-Control-Impact design with temporal pairing (BACI-P) and spatial replication at both control (non-invaded) and impact (invaded) sites (average replication per year in invaded sites = 204, average in non-invaded sites = 237). Spatially replicated BACI-P experiments are the most effective means of assessing environmental impacts on populations in the real, but variable, world^19^,^20^. We predicted that lionfish-induced reductions in tomtate abundance were minor up to the early 2000s (when lionfish abundances were low), significantly large by the late 2000s (when lionfish were relatively abundant), and even larger by the early 2010s (when lionfish reached their highest abundances to date).

## Results

**Figure 1:**
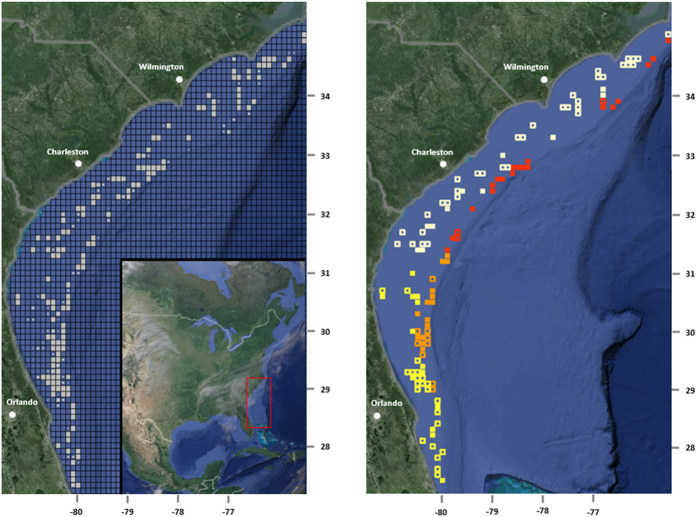
Lionfish range during 2011–2014. (**A**) Southeast Reef Fish Survey video sampling sites in Atlantic water off the southeastern coast of the United States. Grid squares are 1/10^th^ of a degree latitude by 1/10^th^ of a degree longitude (approximately 11 km × 11 km squares). Gray square size indicates the number of samples taken in each grid square. Gray squares that fill the entire grid square indicate squares sampled ten or more times. (**B**) Invaded (filled in squares) and non-invaded (non-filled in squares) areas in 4 regions of the Atlantic off of the southeastern coast of the United States. The northern near-coast region (white squares) was 23.9% invaded (11/46 squares), the northern off-coast region (red squares) was 87.5% invaded (21/24 squares), the southern near-coast region (bright yellow squares) was 37.1% invaded (13/35 squares), and the southern off-coast region (orange squares) was 60.9% invaded (14/23 squares). The maps were generated using ‘maps’ and ‘ggmap’ libraries, available in R 3.1.3 (R: A Language and Environment for Statistical Computing, R Core Team, R Foundation for Statistical Computing, Vienna, Austria (2015) https://www.R-project.org).

One hundred and twenty-eight 11 km × 11 km squares were sampled 10 or more times off of the SEUS (Fig. 1A). The proportion of area invaded was highest in the northern off-shore region (21/24 squares, 87.5%), second highest in the southern off-shore region (14/23, 60.9%), third highest in the southern near-shore region (13/35, 37.1%), and lowest in the northern near-shore region (11/46, 23.9%; Fig. 1B). The results of the tomtate standardization analysis (zero-inflated negative binomial models) that was conducted to account for potential sampling biases and fluctuations in environmental conditions within invasion areas are outlined in the supplemental information (Supplementary Fig. 1).

**Figure 2:**
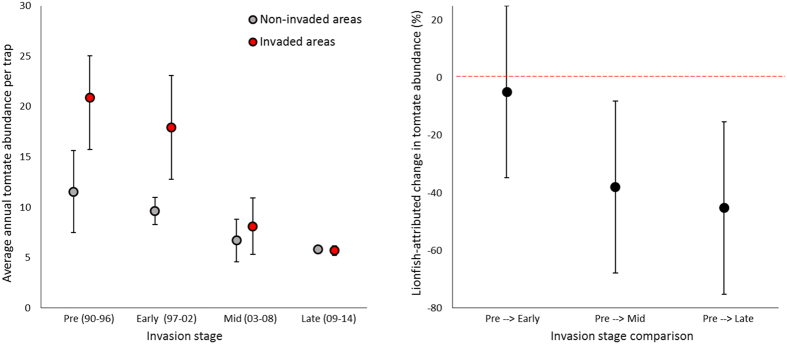
Impact of invasive lionfish on tomtate abundance. (**A**) Average standardized tomtate abundance per trap at different stages of the lionfish invasion for areas not invaded by lionfish (gray points) and areas invaded by lionfish (red points). (**B**) Lionfish-attributed change in tomtate abundance from the pre-invasion stage (1990–1996) up to the early invasion stage (1997–2002), the mid-invasion stage (2003–2008), and the late invasion stage (2009–2014). The red horizontal line indicates no change. Error bars are 95% confidence intervals. Results apply to hard-bottom reef habitat in Atlantic waters from North Carolina to Florida.

Our spatially replicated BACI-P analysis revealed that during the pre-invasion stage (1990–1996), average tomtate abundance was 20.9 tomtate per trap (95% CI = 16.7–25.0, N = 7) in invaded areas and 11.5 tomtate per trap (95% CI = 7.5–15.6, N = 7) in non-invaded areas (Fig. 2A). By the late invasion stage (2009–2014), average tomtate abundance dropped to 5.7 tomtate per trap (95% CI = 5.2–6.2, N = 6) in invaded areas and 5.8 tomtate per trap (95% CI = 5.5–6.2, N = 6) in non-invaded areas (Fig. 2A). Thus, while average tomtate abundance declined over the entire course of the time series in both invaded and non-invaded areas, the significantly larger reduction in invaded areas (interaction term from mixed-effects ANOVA *p* = 0.009) indicates lionfish have had an impact on tomtate abundance. Specifically, the mixed-effects ANOVA revealed that invaded areas experienced a 4.9% greater reduction (*p* = 0.750) than non-invaded areas by the early-invasion stage (1997–2002), a 38.0% greater reduction (*p* = 0.017) by the mid-invasion stage (2003–2008), and a 45.3% greater reduction (*p* = 0.005) by the late invasion stage (Fig. 2B).

## Discussion

Lionfish densities now exceed those of all but the most common predatory fish species on many reefs off of the SEUS, Caribbean, and Gulf of Mexico, and as such, it is imperative to understand how invasive lionfish affect native fish species. Our study is the first to examine the impact of the lionfish invasion on native fish abundance on a broad regional scale and over the entire duration of the lionfish invasion. Our results suggest that 1) lionfish-impacted areas off of the southeastern United States are most prevalent off-shore near the continental shelf-break but are also common near-shore and 2) in impacted areas, lionfish have reduced the pre-invasion tomtate abundance by 45%. Our result of a 45% reduction corroborates the results of experimental manipulations and, thus, suggests that 1) small-scale manipulative studies on patch reefs in localized areas of the Bahamas may well indicate the region-wide impact of the lionfish invasion and 2) our results with tomtate may indicate the impact of the lionfish invasion on native fish abundance in general^10^,^14^.

The lionfish-attributed reduction in tomtate abundance that we observed appears most likely to be explained by reduced survivorship to the adult stage via lionfish predation on juveniles (increased juvenile mortality). Tomtate reach maturity at a fork length of approximately 14 cm^17^, which is too large a size to be common prey for lionfish (lionfish max prey size is usually identified as 10–13 cm^18^,^21^. Consequently, as 99.8% of tomtate sampled in our study were 14 cm or greater, lionfish must be reducing the number of adults via predation on juveniles that in turn reduces survivorship to the adult stage. This mechanism could potentially cause a similar effect in economically important species such as snappers and groupers and potentially even a larger effect on smaller-sized species that are susceptible to lionfish predation over their entire lifespan. Additionally, snappers and groupers may compete with lionfish for food and, as a result, lionfish may further affect snappers and groupers.

The change in the lionfish impact over the different time blocks/invasion stages was in line with our hypothesis that steady increases in lionfish abundance in invaded areas since the late 1990s would cause a steady decline in tomtate abundance. However, the lionfish impact did not increase from the mid-invasion stage to the late invasion stage by as much as might be expected. One possible explanation for this finding is that lionfish predation on juvenile tomtate is frequency-dependent, where lionfish preferentially consume juvenile tomtate when they are common but switch to other prey when juvenile tomtate become relatively rare^22^,^23^. Another possible explanation for this finding is that juvenile tomtate survival to the adult stage is density-dependent. Thus, while increases in lionfish abundance from the mid- to the late-invasion stage may have further reduced the abundance of juvenile tomtate, part of the reduction may not have carried over to the adult stage, which is the stage we sampled^24^,^25^.

To estimate the impact of the lionfish invasion on tomtate abundance, we employed a spatially replicated BACI-P design in which we separated samples into invaded (impacted) and non-invaded (control) sites. By employing this design, any reduction in tomtate abundance that occurred in both invaded and non-invaded areas (which turned out to be a significant reduction) was implicitly assumed to be due to factors other than the lionfish invasion (e.g., climate change, fishing pressure). However, the lionfish invasion has likely had at least some negative impact on tomtate abundance in non-invaded areas. Tomtate are broadcast spawners and their eggs are carried on ocean currents^16^. As a result, there is likely considerable dependency in tomtate abundance between invaded and non-invaded areas. Consequently, any negative impact of the lionfish invasion on tomtate abundance in invaded areas would likely carry over and also impact tomtate abundance in non-invaded areas. If the lionfish invasion has negatively impacted tomtate abundance in non-invaded areas, it also means that lionfish have had a larger impact in invaded areas than our estimate suggests (due to the method we used to estimate the impact of the lionfish invasion). Thus, the lionfish impact on tomtate abundance in invaded Atlantic waters that we report here should be viewed as a conservative estimate.

Nonetheless, it is possible that our estimate of the lionfish impact on tomtate abundance is inflated. While a spatially replicated BACI-P study design (such as the one employed in our study) is the most effective means of assessing environmental impacts on wild populations^19^,^20^, it is not a manipulative experiment. Consequently, it is possible that an unmeasured variable may be the true, underlying cause of the reduction in tomtate abundance that we attributed to the lionfish invasion. However, if a confounding factor is responsible for some or all of the reduction in tomtate abundance that we attributed to lionfish, it must be a variable outside of those that we accounted for in our standardization models (time of year, time of day, location, depth, presence of a non-lionfish predator, bottom temperature, and trap soak time), it must vary in concert with the presence of lionfish, and it must have steadily changed over the past two decades to cause the differential impact we observed from the early invasion stage to the late invasion stage. While it is possible that one or more native predators may have similar habitat preferences as lionfish, no native predator of tomtate or combination of native predators (e.g., black sea bass (*Centropristis striata*), red snapper (*Lutjanus campechanus*), red grouper (*Epinephelus morio*), scamp (*Mycteroperca phenax*)) is known to have experienced changes in abundance that would be expected to cause the differential impact we observed from the early stage to the late stage^26^. Thus, we believe it is unlikely that confounding factors are the true cause of some or all of the reduction in tomtate abundance that we attributed to lionfish.

The majority of lionfish control efforts to date have focused on manual removal^27^, as lionfish are not effectively targeted by standard fishing gears such as fish traps or hook-and line. One study that performed targeted local removals of lionfish over a 7-month span reduced the abundance of lionfish and shifted the composition of lionfish populations to smaller size classes^28^. A second study on the effectiveness of lionfish removals found that 2 years after lionfish removals began, lionfish abundance was 4.2 times lower in areas where lionfish were manually removed than in areas where they were not^29^. Thus, manual removals appear to be effective at least over localized spatial areas and over short durations. However, recent modeling results suggest that sporadic, infrequent lionfish removal events that leave remnant populations are not effective at curbing lionfish biomass over a temporal span of greater than 5 years^11^. Further, lionfish removal programs are often conducted at depths less than 20 meters, while our results indicate that lionfish are most abundant in deeper water. Regardless of depth, the area over which lionfish are distributed in Atlantic waters off the SEUS is sufficiently large (tens of thousands of km^2^) that regional reduction or eradication strategies based on removals are impractical. Therefore, an alternative control strategy would be needed to successfully combat the effect of invasive lionfish on native reef species. Barring the development of a control strategy that reverses the lionfish invasion, lionfish will likely stay at or above their current abundance in the Atlantic, Caribbean, and Gulf of Mexico. Consequently, lionfish will likely continue to suppress native fish abundance for the foreseeable future.

## Materials and Methods

### Data source

To characterize the impact of the lionfish invasion on tomtate abundance in Atlantic waters off of the SEUS, we analyzed fishery-independent data collected by three separate sampling programs that share a single standardized sampling protocol. The Marine Resources Monitoring, Assessment, and Prediction (MARMAP) program of the South Carolina Department of Natural Resources has used chevron traps to monitor reef fish abundance since the late 1980s^30^. Since 2009, MARMAP sampling has been supplemented by the cooperative Southeast Area Monitoring and Assessment Program – South Atlantic. We also included in our analyses 2010–2014 data from the Southeast Fishery-Independent Survey, which the U.S. National Marine Fisheries Service created in 2010 to increase fishery-independent sampling in the SEUS. Hereafter, the three sampling programs are referred to as the Southeast Reef Fish Survey (SERFS). The SERFS data collection activities were approved through various Scientific Research Permits issued by NOAA and other permitting bodies. Collected samples were utilized in accordance with the guidelines of the US Government Principles for the Utilization and Care of Vertebrate Animals Used in Testing, Research, and Training.

The SERFS deploys baited traps in water between approximately 15 and 100 meters deep from Cape Hatteras, North Carolina, to St. Lucie Inlet, Florida^31^. Sampling sites are selected at random from a database of known hard-bottom habitat sites. Traps are typically deployed in groups of six and each trap in a group is deployed at least 200 meters from all other traps. The quantity of each fish species caught, year, date, time of day, latitude, longitude, depth, bottom temperature, and trap soak time are recorded for each sample. The SERFS began attaching high-definition video cameras to all chevron traps in 2011. Video reading commences 10 minutes after a trap lands on the bottom to allow time for the trap to settle. For each video sample, one-second snapshots are read every 30 seconds for 20 minutes, totaling 41 snapshots read. The abundances of a suite of fish species (including lionfish) are then quantified as the sum of the number of individuals in the species that are seen across all 41 frames^32^.

### Before-after-control-impact classification

To determine control and impact areas for our spatially replicated BACI-P analysis, we first partitioned the SERFS sampling range into squares of 1/10^th^ of a degree latitude by 1/10^th^ of a degree longitude (approximately 11 km × 11 km squares). We then used video data (from 2011–2014) to determine control (non-invaded) and impact (invaded) squares. Videos were used to determine which squares were occupied by lionfish instead of trap data because traps have a lower lionfish detection rate (likely due to lionfish preference for live prey, and thus lack of attraction to the baited traps). After removing samples with fewer than 41 video frames and those with unusual trap soak times (very long soak times may indicate an issue with the sample), 3,963 random video samples were available for analysis.

To account for a potentially low lionfish detection rate per video (though still higher than a trap’s), only squares with 10 or more video samples (128 squares) were included in the analysis. Squares with greater than 10 samples were then randomly sub-sampled so that each square contained 10 samples. The sampling intensity of each square was set to a constant value so that our survey’s detection rate would be the same across all squares. If a lionfish was seen on one or more of the 10 video samples within a square, the square was classified as impacted/invaded. Conversely, squares in which no lionfish were seen were designated as control/non-invaded squares.

We set 1990–1996 as the period “before impact” (pre-invasion stage). Although lionfish are thought to have been introduced into Atlantic waters off of the SEUS by the early 1990s, we assumed lionfish abundance was near 0 in both invaded and non-invaded areas prior to 1997. We then partitioned the time period after 1996 into three distinct “after impact” stages; early invasion (1997–2002), mid-invasion (2003–2008), and late invasion (2009–2014). We assumed that the average lionfish abundance in the non-invaded (control) area stayed near 0 throughout all three “after impact” invasion stages. Conversely, based on the results of observational studies of lionfish densities^8^,^9^,^33^ and anecdotal sightings of lionfish^2^,^3^, we assumed that the average lionfish abundance across the invaded (impacted) area increased slightly from the pre-invasion stage to the early-invasion stage, that it increased substantially from the pre-invasion stage to the mid-invasion stage, and that it further increased from the pre-invasion stage to the late-invasion stage. Consequently, we predicted that lionfish would have a differential impact on tomtate abundance across the invasion stages, with only a small impact occurring by the early-invasion stage, a moderate impact occurring by the mid-invasion stage, and the largest impact occurring during the late-invasion stage.

### Tomtate standardization

Even though our sampling was designed to be random, samples within each invasion area may have varied across years with respect to some spatial or temporal pattern of sampling or with fluctuations in environmental conditions^34^. Any such pattern could potentially confound our results if it also affected tomtate abundance (e.g., invaded samples were made in shallower water during early years and deeper water during later years and depth affects tomtate abundance). Consequently, before conducting our BACI-P analysis, we employed zero-inflated negative binomial models (ZINB) to standardize tomtate abundance within each invasion area. Zero-inflated models are valuable tools for modeling distributions that do not fit a standard error distribution due to an excessive number of zeroes^35^. These data distributions are often referred to as “zero-inflated” and are a common condition of count-based ecological data. Due to the high proportion of zero counts found in our data set, we used a zero-inflated mixed model approach that models the occurrence of zero values using two different processes, a binomial process and a negative binomial count process^35^.

The main goal of the ZINB standardization analysis within each area was to standardize the yearly catch of tomtate by extracting the year effects of the model at average values of all other predictor variables^36^. The extracted year effects from each ZINB were then used in the subsequent mixed-effects ANOVA. The same ZINB model was applied for invaded and non-invaded areas:


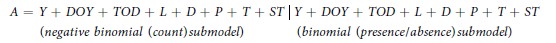


where 

*A* = Tomtate abundance per trap (Count)

*Y* = Year (Common Era)

*DOY* = Day of year (Julian day)

*TOD* = Time of day (24 hour clock)

*L* = Location (Latitude and proximity to continental shelf break)

*D* = Depth (Meters)

*P* = Grouper or snapper predators (Presence/absence)

*T* = Bottom temperature (Celsius)

*ST* = Soak time (Minutes)

To allow for potential non-linear relationships between the predictor variables and tomtate abundance (and to improve model convergence), each continuous predictor variable was converted into a categorical variable. Each year was treated as a separate category. All other continuous predictor variables were separated into quantiles. The location variable had four categories that were developed based on latitude and proximity to the continental shelf break; the northern off-shore region near the shelf break (latitude > 31.25° and longitude < 3/10^ths^ of a degree off the shelf break), the northern near-shore region (latitude > 31.25° and longitude > 3/10^ths^ of a degree off the shelf break), the southern off-shore region near the shelf break (latitude < 31.25° and longitude < 3/10^ths^ of a degree off the shelf break), and the southern near-shore region (latitude < 31.25° and longitude > 3/10^ths^ of a degree off the shelf break). The snapper/grouper variable had two categories that were based on the presence/absence of 7 species; speckled hind (*Epinephelus drummondhayi*), red grouper (*Epinephelus morio*), warsaw grouper (*Epinephelus nigritus*), snowy grouper (*Epinephelus niveatus*), red snapper (*Lutjanus campechanus*), gag (*Mycteroperca microlepis*), and scamp (*Mycteroperca phenax*). If any one of these 7 species were present in a trap, snappers/groupers were designated as being present. After removing samples that fell outside of squares classified as invaded or non-invaded and samples missing data on any of the predictor variables included in our analyses, we were left with 5,088 samples from the invaded area and 5,926 samples from the non-invaded area (Supplementary Table 1).

### Mixed-effects ANOVA

The year effects for each invasion area (invaded and non-invaded) that we obtained from the ZINB standardization models were used to conduct the mixed-effects ANOVA. First, each year effect from each invasion area was assigned an invasion stage (pre-invasion, early-invasion, mid-invasion, and late-invasion). We then conducted a mixed-effects ANOVA (year was a random effect) on invasion stage, invasion area, and the interaction between invasion stage and invasion area. The mixed-effect ANOVA allowed us to partition effects on mean tomtate abundance into area effects that may have existed between invaded and non-invaded sites (e.g., potential differences in habitat quality or depth), temporal effects that may have changed over time blocks (e.g., climate change, fishing), and interaction effects between areas and time blocks (i.e., the differential impact of lionfish across invasion stages). The interaction effects that indicated the impact of the lionfish invasion (in invaded areas) on tomtate abundance from the pre-invasion stage up to each subsequent invasion stage were the primary focus of this study.

### Other native fish species

After completing our analysis of the lionfish impact on tomtate, we explored if we could conduct the same analysis with other native fish species caught by the SERFS. To conduct our analysis, species would need to be relatively abundant in both invaded and non-invaded areas prior to the onset of the lionfish invasion (pre-1997). Unfortunately, none of the species investigated (other than tomtate) exhibited such a pattern in abundance. Specifically, white grunt (*Haemulon plumieri)*, black sea bass (*Centropristis striata*), and *Stenotomus spp*. had low abundances across the invaded area before the lionfish invasion began. Conversely, vermilion snapper (*Rhomboplites aurorubens*) had a low abundance in the non-invaded area before the lionfish invasion began. Consequently, it was not possible to compare, in a statistically rigorous manner, changes in abundance in the invaded area relative to the non-invaded area for any of the species investigated. Thus, the impact of lionfish on other native fish species was not explored further in this study.

## Additional Information

**How to cite this article**: Ballew, N. G. *et al*. Invasive lionfish reduce native fish abundance on a regional scale. *Sci. Rep.* **6**, 32169; doi: 10.1038/srep32169 (2016).

## Supplementary Material

Supplementary Information
