# Supplementary material for: Invasive lionfish reduce native fish abundance on a regional scale
Source: Sci Rep. 2016 Aug 31;6:32169. doi: 10.1038/srep32169 (PMC5005992; doi:10.1038/srep32169)
Supplement: Supplementary Information [file srep32169-s1.doc]

Invasive lionfish reduce native fish abundance on a regional scale

Supplementary Information

Nicholas G. Ballew*, Nathan M. Bacheler, G. Todd Kellison, and Amy M. Schueller

National Marine Fisheries Service, Southeast Fisheries Science Center, Beaufort Laboratory,

101 Pivers Island Road, Beaufort, NC 28516 USA

|  | Samples | | Mean Day  (Julian Day) | | Mean TOD  (24 Hour Clock) | | Location  (% for dom. loc.) | | Other Predators  (% w/predators) | | Mean Depth  (Meters) | | Mean Temp  (Celsius) | | Mean Soak  (Minutes) | |
| --- | --- | --- | --- | --- | --- | --- | --- | --- | --- | --- | --- | --- | --- | --- | --- | --- |
| Year | INV | NON | INV | NON | INV | NON | INV | NON | INV | NON | INV | NON | INV | NON | INV | NON |
| 1990 | 109 | 174 | 169.2 | 133.6 | 16.4 | 16.4 | 45.0 | 100.0 | 31.2 | 8.6 | 43.8 | 26.4 | 23.0 | 21.2 | 104.6 | 101.2 |
| 1991 | 93 | 149 | 218.3 | 214.2 | 15.8 | 17.3 | 55.9 | 98.0 | 23.7 | 10.1 | 43.8 | 27.4 | 24.3 | 25.3 | 93.5 | 89.6 |
| 1992 | 98 | 181 | 148.1 | 155.0 | 16.7 | 16.6 | 72.5 | 98.3 | 25.5 | 8.3 | 44.4 | 28.4 | 21.3 | 21.2 | 96.9 | 100.3 |
| 1993 | 160 | 217 | 164.0 | 183.8 | 16.6 | 17.2 | 66.3 | 98.6 | 28.8 | 6.5 | 46.8 | 26.5 | 21.0 | 24.0 | 97.4 | 99.1 |
| 1994 | 187 | 184 | 168.1 | 183.8 | 16.3 | 16.9 | 53.5 | 97.8 | 38.0 | 8.7 | 48.4 | 27.8 | 22.4 | 23.4 | 99.8 | 102.4 |
| 1995 | 138 | 225 | 193.6 | 202.0 | 15.4 | 15.2 | 48.6 | 98.2 | 40.6 | 2.7 | 48.2 | 25.9 | 23.5 | 25.2 | 99.1 | 99.5 |
| 1996 | 233 | 192 | 181.0 | 186.6 | 15.9 | 17.4 | 58.4 | 98.4 | 31.3 | 5.2 | 47.9 | 27.4 | 21.3 | 22.6 | 101.9 | 97.7 |
| 1997 | 205 | 196 | 195.1 | 182.7 | 15.9 | 16.5 | 52.7 | 78.1 | 38.5 | 13.8 | 49.0 | 30.7 | 22.6 | 22.6 | 99.7 | 96.7 |
| 1998 | 155 | 242 | 145.5 | 188.9 | 14.6 | 16.8 | 52.3 | 67.8 | 26.5 | 12.8 | 47.5 | 35.1 | 20.6 | 20.7 | 102.5 | 96.5 |
| 1999 | 103 | 110 | 213.0 | 190.6 | 16.5 | 16.7 | 59.2 | 77.3 | 34.0 | 12.7 | 47.9 | 28.5 | 22.0 | 23.5 | 100.3 | 97.2 |
| 2000 | 155 | 150 | 194.3 | 201.8 | 16.4 | 15.7 | 52.3 | 90.0 | 31.6 | 16.0 | 44.9 | 26.8 | 23.8 | 24.4 | 100.9 | 99.9 |
| 2001 | 123 | 113 | 199.9 | 207.3 | 15.6 | 14.9 | 71.5 | 80.5 | 29.3 | 23.9 | 45.5 | 29.3 | 23.1 | 24.1 | 95.5 | 100.8 |
| 2002 | 133 | 152 | 191.4 | 215.3 | 16.6 | 17.0 | 30.8 | 67.1 | 27.8 | 28.3 | 46.6 | 32.7 | 22.7 | 25.5 | 102.0 | 98.9 |
| 2003 | 137 | 109 | 206.6 | 191.4 | 16.5 | 15.4 | 64.2 | 68.8 | 19.7 | 18.4 | 47.5 | 30.2 | 18.5 | 19.3 | 101.8 | 101.4 |
| 2004 | 143 | 154 | 165.7 | 182.0 | 16.3 | 16.1 | 58.7 | 71.4 | 24.5 | 24.7 | 46.7 | 34.7 | 19.6 | 21.9 | 97.4 | 97.6 |
| 2005 | 130 | 153 | 201.0 | 184.1 | 15.9 | 15.4 | 50.0 | 69.3 | 23.1 | 16.3 | 44.6 | 34.0 | 23.4 | 22.7 | 97.9 | 98.5 |
| 2006 | 130 | 148 | 203.2 | 200.3 | 15.6 | 16.0 | 69.2 | 75.0 | 13.1 | 15.5 | 46.1 | 32.3 | 21.1 | 23.3 | 92.0 | 93.7 |
| 2007 | 157 | 168 | 198.4 | 203.9 | 15.4 | 16.6 | 60.5 | 75.0 | 28.7 | 13.7 | 45.4 | 31.3 | 22.5 | 24.1 | 93.8 | 92.7 |
| 2008 | 151 | 154 | 206.1 | 180.0 | 16.4 | 16.1 | 57.6 | 81.2 | 12.6 | 11.7 | 46.3 | 29.9 | 21.8 | 21.6 | 93.9 | 92.9 |
| 2009 | 169 | 226 | 207.5 | 194.5 | 15.3 | 16.6 | 49.7 | 67.3 | 10.1 | 11.1 | 45.0 | 33.6 | 22.5 | 22.1 | 96.0 | 94.7 |
| 2010 | 317 | 382 | 237.7 | 234.4 | 15.9 | 16.5 | 33.8* | 46.1 | 12.6 | 15.2 | 42.4 | 36.0 | 23.5 | 22.2 | 100.2 | 98.1 |
| 2011 | 311 | 393 | 216.1 | 198.6 | 15.8 | 15.2 | 34.4 | 35.4 | 12.5 | 17.6 | 45.3 | 37.7 | 22.1 | 21.2 | 96.8 | 98.9 |
| 2012 | 507 | 594 | 200.9 | 189.3 | 15.7 | 15.8 | 48.9 | 59.6 | 19.7 | 23.1 | 48.3 | 35.7 | 21.6 | 22.2 | 95.0 | 93.6 |
| 2013 | 575 | 670 | 194.2 | 196.7 | 15.3 | 15.6 | 35.5 | 45.8 | 14.1 | 18.2 | 44.0 | 33.9 | 21.2 | 22.1 | 96.4 | 94.6 |
| 2014 | 469 | 490 | 182.9 | 190.5 | 15.6 | 15.5 | 43.3 | 49.8 | 14.3 | 19.8 | 46.3 | 34.3 | 22.5 | 23.3 | 94.2 | 92.0 |
| AVE | 204 | 237 | 192.1 | 191.7 | 15.9 | 16.2 | 52.7 | 75.8 | 24.5 | 14.5 | 46.1 | 31.1 | 22.1 | 22.8 | 98.0 | 97.1 |

Supplementary Table 1. Summary statistics for samples taken from 1990 – 2014. The number of samples taken annually in each range (INV = invaded; NON = non-invaded) are depicted. For continuous variables, the mean value is reported. For categorical variables, percentages of categories are reported. The dominant location of sampling each year for non-invaded areas was in the north near the coast and for invaded areas it was in the north near the continental shelf-break. * the dominant location for sampling in invaded areas in 2010 was in the south near the shelf-break.


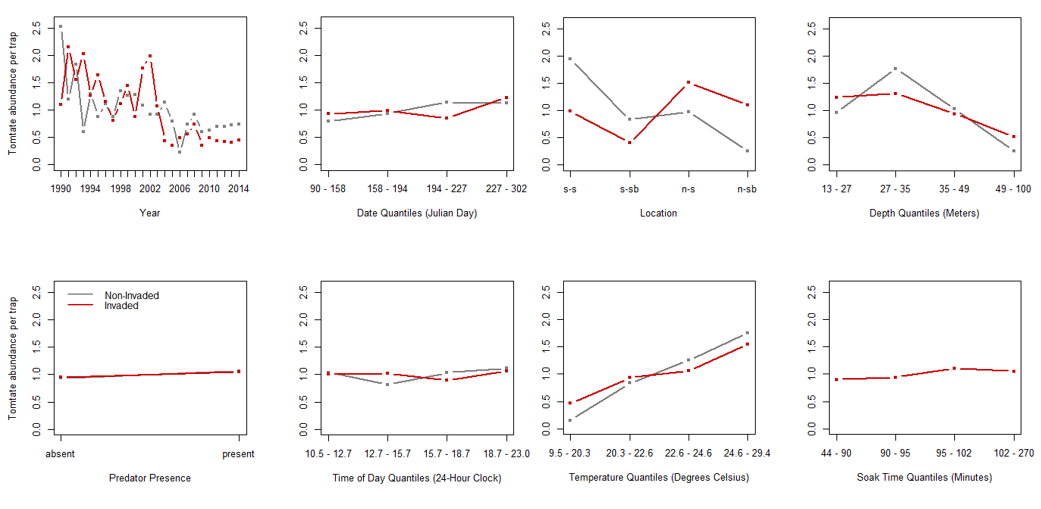


Supplementary Figure 1. Results of the zero inflated negative binomial model data standardization analysis for each area. Relationship between predictor variables and tomtate abundance per site in areas not invaded (gray lines) and invaded (red lines) by lionfish. Tomtate abundance is depicted relative to the average abundance across the variable. Samples were categorized by quantile prior to being separated into invasion categories. Thus, the quantile categories contain the same environmental values across invasion categories. For the location variable, s-s is the south region near the coast, s-sb is the south region near the continental shelf-break, n-s is the north region near the coast, and n-sb is the north region near the continental shelf-break.
